# Assessing Dutch women’s experiences of labour and birth: adaptations and psychometric evaluations of the measures Mothers on Autonomy in Decision Making Scale, Mothers on Respect Index, and Childbirth Experience Questionnaire 2.0

**DOI:** 10.1186/s12884-022-04445-0

**Published:** 2022-02-18

**Authors:** L. L. Peters, M. S. G. van der Pijl, S. Vedam, W. S. Barkema, M. T. van Lohuizen, D. E. M. C. Jansen, E. I. Feijen-de Jong

**Affiliations:** 1 Department of General Practice & Elderly Medicine, University Medical Center Groningen, University of Groningen, Groningen, The Netherlands; 2 Amsterdam UMC, Vrije Universiteit Amsterdam, Department of Midwifery Science AVAG, Amsterdam Public Health Research Institute, Amsterdam, The Netherlands; 3 AVAG (Midwifery Academy Amsterdam Groningen), Groningen, The Netherlands; 4 Birth Place Laboratory, Division of Midwifery, University of British Columbia, Vancouver, BC Canada

**Keywords:** Childbirth, Personal autonomy, Decision making. Respect, Patient reported outcome measure, Psychometrics, Midwifery, Obstetrics

## Abstract

**Background:**

The Mothers Autonomy in Decision Making Scale (MADM) assesses women’s autonomy and role in decision making. The Mothers on Respect Index (MORi) asseses women’s experiences of respect when interacting with their healthcare providers. The Childbirth Experience Questionnaire 2.0 assesses the overall experience of childbirth (CEQ2.0). There are no validated Dutch measures of the quality of women’s experiences in the intrapartum period. Therefore, the aim of this study was to evaluate the psychometric properties of these measures in their Dutch translations.

**Methods:**

The available Dutch versions of the MADM and MORi were adapted to assess experiences in the intrapartum period. The CEQ2.0 was translated by using forward-backward procedures. The three measures were included in an online survey including items on individual characteristics (i.e. maternal, birth, birth interventions). Reliability was assessed by calculating Cronbach’s alphas. Mann-Whitney, Kruskal Wallis or Student T-tests were applied where appropriate, to assess discrimination between women who differed on individual characteristics (known group validity). We hypothesized that women who experienced pregnancy complications and birth interventions would have statistically lower scores on the MADM, MORi and CEQ2.0, compared with women who had healthy pregnancies and physiological births. Convergent validity was assessed using Spearman Rank correlations between the MADM, MORi and/or CEQ2.0. We hypothesized moderate to strong correlations between these measures. Women’s uptake of and feedback on the measures were tracked to assess acceptability and clarity.

**Results:**

In total 621 women were included in the cross sectional study. The calculated Cronbach’s alphas for the MADM, MORi and CEQ, were ≥ 0.77. Knowngroup validity was confirmed through significant differences on all relevant individual characteristics, except for vaginal laceration repair. Spearman Rank correlations ranged from 0.46-0.80. In total 98% of the included women out of the eligible population completed the MADM and MORi for each healthcare professional they encountered during childbirth. The proportions of MADM and MORi-items which were difficult to complete ranged from 0.0-10.8%, 0.6-2.7%, respectively.

**Conclusions:**

The results of our study showed that the Dutch version of the MADM, MORi and CEQ2.0 in Dutch are valid instruments that can be used to assess women’s experiences in the intrapartum period.

**Supplementary Information:**

The online version contains supplementary material available at 10.1186/s12884-022-04445-0.

## Background

In the last decade, there is increasing attention to facilitating autonomy and respect during childbirth as a core indicator of quality care [1, 2]. This emphasis is also stated in guidelines of the World Health Organization (2018). “Women’s experience of care is a priority and even when a medical intervention is wanted or needed, the inclusion of women in making decisions about the care they receive is important to achieve a positive childbirth experience” [3]. Maternal healthcare providers’ influence on autonomy and therefore women’s participation in decision making is substantial [4]. Respectful maternity care is an approach to care that emphasizes the right of women, infants and their families to receive evidence based care while taking into account their personal needs and preferences [5–7]. The White Ribbon Alliance delinated seven essential Respectful Maternity Care rights including: the right to information, informed consent and refusal (respect for her choices and preferences), the right to liberty, autonomy, self/determination and freedom from coercion and the right to be treated with dignity and respect [8].

In the Netherlands a questionnaire study performed in 2016 among 2377 women showed that 92% of women report ‘good to best’ possible care during labour and birth [9, 10], whereas 8% of women reported their care experience as “less than good” [9]. In 2017, the perceptions and views of 2192 women with a self reported traumatic birth experience were reported. This study revealed that lack of autonomy is one of the leading causes of a traumatic birth experience among Dutch women. According to the women, the traumatic experience could have been prevented by better communication and support of maternal healthcare providers during labour and birth, which are important aspects of Respectful Maternity Care [11]. In 2018, a cross sectional study was conducted among 557 women to assess their experiences in the prenatal period; 83% reported experiences of high respect, and 62% experienced high autonomy [12]. Furthermore, studies show that individual characteristics, such as age, ethnicity, education level and parity, are associated with the quality of women’s birth experience and perception of care, substantiating the need for attention on different groups of women [13–15]. Although women’s experiences are important during every stage of pregnancy, it is especially relevant during labour and birth due to the intensity and vulnerability of giving birth as a major life event. At the same time, unexpected intrapartum events can lead to situations in which women’s autonomy and control is under pressure [16, 17].

Dutch maternity care is divided into midwife-led care and obstetrician-led care. Under midwife-led care, pregnant women at low risk of complications are cared for by autonomous midwives in the community, throughout the prenatal, intrapartum and postpartum periods. During birth, midwives are assisted by trained maternity care assistants. Birth takes place at home, or at a birth center (separately or (next to) a hospital). When complications occur in pregnancy, during or right after birth, or when pharmacological pain relief is requested during birth, women are referred to obstetrician-led care. Obstetrician-led care takes place in the hospital where women are cared for by hospital based-midwives and residents under oversight of an obstetrician, and assisted by obstetric nurses. When the clinical situation indicates a need for specialist expertise or surgery, obstetricians provide direct care [18].

Measures have been developed to assess women’s autonomy and respect when accessing perinatal health care. The valid Mothers Autonomy in Decision Making Scale (MADM), assesses women’s autonomy and role in decision making during maternity care [19]. The Mothers on Respect Index (MORi) measures women’s experiences of respect when interacting with their maternity healthcare providers [20]. In Canada, both measures were found reliable and valid to measure women’s autonomy and respect during pregnancy, childbirth and postpartum phases of care [19, 20]. The MORi and MADM were translated and adapted to the Dutch maternity care system [12]. Both measures were evaluated with as having good psychometric properties to assess the experienced autonomy and respect among Dutch pregnant women in the prenatal period; however, properties have yet to be assessed in the intrapartum period [12]. Currently, it is unknown if Dutch women report MADM and MORi scores differently based on birth characteristics (e.g. mode of birth, place of birth) and intrapartum interventions (e.g. induction, episiotomy, pain relief treatment). Another validated measure that assesses the overall experience of childbirth is the Childbirth Experience Quesionnaire 2.0 (CEQ 2.0) [21–23]. To date, the CEQ2.0 has been translated and evaluated psychometrically in settings in Sweden, United Kingdom and Iran [21–23]. Until now a Dutch version of the CEQ2.0 was not available.

Valid measures to assess women’s autonomy, respect and overall childbirth experience are highly relevant for research purposes as well as for clinical settings. Measured experiences can be used as input to develop and optimize maternal care. Therefore this study aims to translate the CEQ2.0 into the Dutch language and evaluate the psychometric properties (reliability and construct validity) of the Dutch versions of the MADM, MORi and CEQ2.0. Since this is the first study that requested women to complete the MADM and MORi for each healthcare provider that attended them in the intrapartum period, we evaluated the acceptiblity and clarity of both measures as well.

## Methods

In order to evaluate the psychometric properties of the Dutch versions of the MADM, MORi and CEQ2.0, we conducted a cross-sectional study in spring 2019All methods were peformed in accordance with the Declaration of Helsinki. We additionnally used the COSMIN checklist to evaluate and report on the psychometric properties of the three measures [24]. In the Netherlands no ethical approval is required regarding this type of research. (http://www.ccmo.nl) The local Medical Research Ethics Committee of the University Medical Center Groningen has confirmed this and defined this study as non-WMO (Medical Research Involving Human Subjects Act, www.ccmo.nl) research (number 2018/185).

### Respondents, inclusion and exclusion criteria

We mounted the survey on an online platform and recruited through social media and networking sampling through midwifery services and organizations. Women could access the survey through a link on Facebook pages of (1) midwifery care practices, (2) postnatal maternity care organisations, (3) all Dutch Midwifery Academies, or (4) through posts on other Facebook pages related to pregnancy and birth. To minimize recall bias, eligibility was limited to women who gave birth < 1 year prior to filling out the survey. Women were included in the analyses, if a community midwife, hospital-based midwife and/or obstetrician provided care in the intrapartum period, and if the MADM, MORi, and CEQ2.0 were completed. The inclusion of women with any combination of maternal healthcare professionals, resulted in participants completing between one or three measures of the MADM and MORi. To avoid collecting data from women who only shared their (most) positive or negative experience, we only included data of women who had completed both measures for all healthcare professionals attending at birth in our analyses for the psychometric properties reliability, known group validity and convergent validity. To assess the psychometric property acceptability, we did included all data without such an exclusion, since it will provide information if women were willing to complete the MADM and MORi multiple times. Women had to be proficient in the Dutch language and had to consent to anonymous use of their data for this study. Women who were 16 years or younger were excluded. We aimed for a sample size of 500 women or more, which is considered ‘very good’ for psychometric purposes [25, 26]. As previous studies reported difficulties in achieving a representative sample regaring ethnicity and socioeconomic position, increased efforts were made to reach these populations by involving midwfiery care practices in areas with a higher density of underrepresented groups [12, 19, 20].

### Measures

In a previous study we translated the MADM and MORi according to WHO-guidelines and adapted both measures to the Dutch healthcare system [12]. Afterwards both measures were evaluated on their psychometric properties [12]. The results of this study supported the feasiblity, reliability and knowngroup validity of the MADM and MORi in pregnant women [12]. For the current study, these Dutch versions were adapted to assess experiences in the intrapartum period and instructions were added to ask women to complete the MADM and MORi separately for each health care provider they encountered in the intrapartum period.

The MADM is a measure that consists of 7 items with responses scored on a 6-point Likert scale ranging from 1 (completely disagree) to 6 (completely agree), resulting in a summed scale score ranging from 7 to 42. A higher score indicates higher experienced autonomy. The psychometric properties of the MADM have been evaluated in Canadian settings on pregnancy and birth experiences of women [19]. In our previous study, the MADM was translated and adapted to the Dutch context and consisted –as the original version- the same number of items and scoring method. Atfterwards the Dutch verions of the MADM was psychometrically evaluated regarding women’s experiences during the prenatal period [12]. In both settings the MADM showed good feasibility and a good internal consistency; Cronbach’s alpha ranged from 0.93-0.96 [12, 19]. In addition, the construct validity of the Dutch MADM showed the ability to discriminate between characteristics of women who differed on demographic characteristics (e.g. Dutch Region), and healthcare provision characteristics (e.g. type of maternal healthcare provider, length of consultations) [12].

The MORi consists of 14 items with responses scored on a 6-point Likert scale ranging from 1 (completely disagree) to 6 (completely agree), resulting in a summed scale score ranging from 14 to 84. The psychometric properties of the MORi have been evaluated based on the experiences of respect that women report on for their entire maternity care journey [20] or during pregnancy care alone [12]. In our previous study, the MORi was translated and adapted to the Dutch context and consisted –as the original version- the same number of items and scoring method. Afterwards the Dutch version of the MORi was psychometrically evaluated regarding women’s experiences during the prenatal period [12]. We reported that the internal consistency of the MORi was satisfactory for the Canadian, USA and Dutch versions i, with Cronbach’s alpha ranging from 0.76 to 0.94 [12, 20]. Additionally, the MORi discriminated between several subgroups based on demographic characteristics (e.g. social economic status, history of substance abuse), pregnancy characteristics (e.g. risk factors present, complications during pregnancy) or healthcare characteristics (type of maternity provider) [12, 20].

The CEQ2.0 contains 22 items and assesses women’s experiences regarding their childbirth across four domains, i.e. own capacity (8 items), perceived safety (6 items), professional support (5 items), and participation (3 items) [21]. The 22 items of the CEQ2.0 were translated from English to Dutch as follows: (1) a forward translation from English to Dutch, by two independent translators of the Language Centre VUmc resulting in two independent translations. (2) Reconciliation of the forward translations by one native English speaker (PdCsenior lecturer/psychologist) and one native Dutch speaker (LLP, author) to one reconciled forward translation. (3) Independent backward translation from Dutch to English, by two Dutch translators fluent in English who were naïve to the measured construct of childbirth experiences. (4) Consensus with a native English speaker (PdC), native Dutch speaker (LLP) and both backward translators, resulting in one final translated version. Since we aimed to include the Dutch version of the CEQ2.0 in an online survey we changed three items that in the original version used a visual analogue scale into a marking scale ranging from 0 to 10. This scale matches the Dutch school marking system, with 0 reflecting the lowest possible score and 10 the highest. Negatively worded items were reversed in scoing and the marking scale-scores were categorized into 0-2 = 1, 3-5 = 2, 6-8 = 3 and 9-10 = 4. The other nineteen items were scored on a 4-point Likert scale ranging from 1 (completely disagree) to 4 (completely agree) [21]. The scoring of the CEQ2.0 is the following, the item ratings per subscale are aggregated to scale scores by summing the coded values of the items in each scale and dividing by the number of items in that subscale [21] The weighted mean CEQ-score can be calculated by adding all subscale scores (own capacity, perceived safety, professional support, and participation) and dividing by four [21]. The theoretical range is from one to four, a higher score indicates more positive experiences [21]. The psychometric properties of the CEQ2.0 had been evaluated based on the childbirth experiences of women one to three months postpartum [21–23]. Internal consistency was satisfactory; Cronbach’s alpha ranged from 0.82-0.90 in different settings in Sweden, United Kingdom, and Iran [21–23]. The CEQ2.0 discriminated between several subgroups based on maternal characteristics (e.g.parity) and birth characteristics (e.g. labour duration) [21]. The Dutch versions of the MADM, MORi and CEQ2.0 are shown in the Additional file 1 including their scoring methods.

### Survey construction

The online survey comprised the Dutch versions of the MADM, MORi and CEQ2.0. Women completed all measures at the same time, and could not skip items. Aditionnally, women provided feedback on the clarity of the MADM and MORi regarding its instructions and on individual items by indicating whether or not these items were difficult to complete (yes/no) [12]. In addition, women were asked if they had any general remarks regarding the completion of the MADM and MORi. Aditionally, we collected data on the following individual demographic characteristics: maternal age, (later categorized into ≤25, 26-30, 31-35, and ≥ 36 years); region, ethnicity, marital status, religion, education, and income. Dutch regions was based on Dutch provinces, North (Groningen, Drenthe, Friesland), East (Overijssel, Gelderland, Flevoland), South (Noord-Brabant, Limburg), and West (Noord-Holland, Zuid Holland, Utrecht, Zeeland). Ethnic background was categorised as no migrant background (Dutch) versus migrant background, which was defined as the women or one of the women’s parents being born in the Dutch Antilles, Aruba, Suriname, Morocco, Turkey, Indonesia, or other foreign country [27]. Marital status at moment of birth was registred as single versus partner/spouse. Religion was measured as none, Christianity, Islam or other. Education level was categorized as follows: Low (i.e. primary school, first three years of secondary school or lower level of vocational training), middle (upper secondary school or medium level of vocational training or work-based learning pathways), or high (higher vocational education, and university education). Family monthly net income was assessed by the following categories < €2000, €2000-€2500, €2501-€3500, ≥ €3501 and the option “I do not want to provide this information” was available.

Women also reported on pregnancy characteristics, including parity (nulliparous or mutliparous), and gestational age, which was categorized in to ≤36 + 6, 37 + 0-39 + 6,40 + 0-40 + 6, 41 + 0-41 + 6, ≥42. Women indicated if they had adverse physical health events during pregnancy measured by yes or no on one of the following complications: intra-uterine growth restriction, blood loss first trimester, blood loss second or third trimester, diabetes gravidarum, cholestasis, problems with blood pressure, abruptio placenta, placenta praevia, polyhydramnion, thrombosis, HELLP-syndrome, dysmature, macrosomia, and/or oligohydramnios. Women indicated if they had adverse psychological health status, by reporting if they had experienced depression or anxiety.

Birth characteristics included mode of birth (vaginal birth, instrumental vaginal birth or caesarean section), the number of times women had met the healthcare provider who attended at their births, in their prenatal period (0, 1-2, 3-4 or ≥ 5 times), place of birth (home, maternity hotel/birth centre, hospital), whether birth interventions took place (i.e. induction of labour, CTG monitoring during labour, pain relief treatment, catheterisation, vaginal laceration repair, vaginal laceration repair that required surgery, and manual placenta removal), and neonatal outcomes (mortality, resuscitation).

We pilot tested the online survey with eight women, after which several technical and content changes were made, for example: information on a couple of items was clarified and explanations on what type of birth interventions took place were adjusted to improve understanding.

### Data analysis

Descriptive statistics were used to describe the maternal, pregnancy and birth characteristics of the included population. Since the MADM and MORi scores distributions were skewed, median scores with corresponding interquartile ranges (IQR) were reported with no decimal places [12]. Women completed an MADM and MORi measure for each involved healthcare provider (community midwife, hospital-based midwife, and obstetrician). Depending on this answer, women completed one to three measures of the MADM and MORi. To report overall median scores of the included population and subgroups who differed on maternal characteristics, we selected at random a MADM or MORi score if women completed two or three measures. The CEQ2.0 scores were reported by weighted mean with standard deviation (SD), the scores were reported with two decimal places [21–23].

Reliability was assessed by calculating Cronbach’s alpha for the MADM, MORi and CEQ2.0. Additionally, the Cronbach’s alphas were calculated for each MADM and MORi specified for the involved midwives, hospital-based midwives and obstetricians. A calculated Cronbach’s alpha of ≥0.70 is satisfactory [28].

Construct validity was assessed in terms of known group validity and convergent validity [29]. Regarding known group validity, we hypothesized similar MADM, MORi and CEQ 2.0 scores on women who differed on demographic characteristics [12]. By comparing those measurement scores with women who had a healthy pregnancy and a physiological birth, statistical significant lower scores of the MADM, MORi and CEQ2.0 would have been reported by women who experienced pregnancy complications, birth interventions (e.g. induction of labour, episiotomy or caesarean section) and adverse neonatal outcomes (i.e. mortality and resuscitation) [12, 17, 19, 20, 30–32]. Statistical differences were calculated on those outcomes if the prevalence was at least 5%. We hypothesized statistical higher scores on the three measures if women received maternal care by a community care midwife, since she can provide continuity of care throughout pregnancy and birth [19–21, 33–36]. Mann-Whitney U, Kruskal Wallis tests, Student T-Tests or Anova Tests were used appropriately, to assess the variability of both measures scores among women that differed on these characteristics.

Next, Spearman Rank correlations were performed to examine convergent validity between the MADM, MORi and CEQ2.0. We expected moderate to strong correlations between the CEQ2.0 and MADM and MORi scores, because respectful maternity care and women’s autonomy are key components of high-quality care during childbirth [37]. We used standard interpretations of the correlation coefficients: 0.40-0.59 moderate; 0.60-0.79 strong and 0.80-1.0 as very strong [38].

As this is the first study requiring women to complete the measures MADM and MORi separately for each health care provider they encountered, the acceptability and clarity of these measures were also assessed. Acceptability refers to the question whether or not women were willing to complete the MADM and MORi multiple times [39]. In our study women indicated how many healthcare providers attended at their births and we observed whether for all of these providers the MADM and MORi were completed. With descriptive statistics the clarity on items on the MADM and MORi as well a general remarks were analysed. All statistical analyses were performed with SPSS Statistics 25.0 (SPSS Inc., Chicago, IL). The level of significance was set at *p* ≤ 0.05.

## Results

**Fig. 1:**
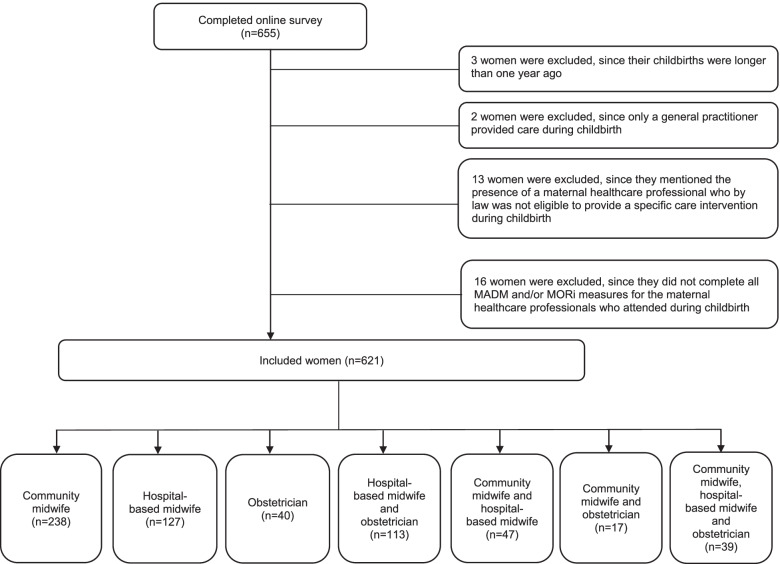
Flowchart of the included study population

In total 655 women completed the online survey in the period March 1st -May 31st 2019. Responses were excluded if the reported birth was longer than one year ago (*n* = 3), and when only a general practitioner was present during childbirth (*n* = 2). Responses were excluded if respondents gave inconsistent answers, e.g. they mentioned the presence of a maternal healthcare provider who by law was not eligible to provide a specific care intervention during childbirth (*n* = 13). Also responses from women who did not complete all measures of the MADM and MORi of all maternal healthcare providers who attended at their labour and birth were excluded (*n* = 16). Of the eligible women who competed the survey, 621 women (95%) were included in the data analyses (Fig. 1 Flowchart of the included study population).

The included respondents accessed the online survey link, published on Facebook pages from community midwifery practices (63.8%), maternity care assistants’ organisations (13.4%), midwifery academies (1.8%), and Facebook pages related to pregnancy and birth (17.6%), and other not further specified Facebook pages (3.4%). The mean age of the included population was 31.2 (SD 4.1) years. The majority of the included women were of Dutch origin (93.6%) and had a partner (98.1%). Fewer women had a low education level (7.6%) and a monthly income less than €2000 (9.8%, Table 1). Among women, 46.5% gave birth for the first time and 96.9% had a delivery at term (gestational age of 37 weeks or more). Of all respondents, 38.3% received care during birth from a community midwife only and 45.1% from a hospital based midwife and/or an obstetrician. In 16.6% of the cases, the respondent was receiving care from a hospital midwife and/or an obstetrician, but the community midwife was also present. Women with a higher education more often received care from both a community midwife and obstetrician (64.1-64.7%) compared to women with a middle education level (28.2-35.3%). Most women who received care primarily by a hospital-based midwife indicated that they had never met them before during their prenatal care (75.6%), while 8.4% of the women receiving care from a primary care midwife had not met them before. Pain relief treatment in the hospital was less common when, next to the other care providers, a community midwife was present (36.2-41.2%) compared to when she was not present (40.2-67.3%). Babies born under care of both a hospital-based midwife and obstetrician showed the highest proportion of resuscitation (11.5%). None of the children died prior, during or shortly after birth (Table 2).

**Table 1:** Maternal characteristics of the included Dutch women for the total population and stratified for the maternal healthcare professional who provided care during labour and birth (*N* = 621)

	Total population	Community midwife	Hospital-based midwife	Obstetrician	Community midwife and Hospital-based midwife	Community midwife and Obstetrician	Hospital-based midwife and Obstetrician	Community midwife, Hospital-based midwife and Obstetrician
	***N = 621*** 100%	***n*** = 238 38.3%	***n*** = 127 20.5%	***n*** = 40 6.4%	***n*** = 47 7.6%	***n*** = 17 2.7%	***n*** = 113 18.2%	***n*** = 39 6.3%
	N (%)	n(%)	n(%)	n(%)	n(%)	n(%)	n(%)	n(%)
**MATERNAL CHARACTERISTICS**								
**Maternal age (in years)**								
≤ 25	44 (7.1)	18 (7.6)	8 (6.3)	3 (7.5)	5 (10.6)	0 (0.0)	9 (8.0)	1 (2.6)
26-30	246 (39.6)	95 (39.9)	45 (35.4)	15 (37.5)	17 (36.2)	7 (41.2)	48 (42.5)	19 (48.7)
31-35	244 (39.3)	90 (37.8)	56 (44.1)	12 (30.0)	22 (46.8)	5 (29.4)	44 (38.9)	15 (38.5)
≥ 36	87 (14.0)	35 (14.7)	18 (14.2)	10 (25.0)	3 (6.4)	5 (29.4)	12 (10.6)	4 (10.3)
**Dutch regions**								
North	256 (41.2)	108 (45.4)	55 (43.3)	15 (37.5)	20 (42.6)	5 (29.4)	40 (35.4)	13 (33.3)
East	144 (23.2)	55 (23.1)	31 (24.4)	8 (20.0)	12 (25.5)	3 (17.6)	29 (25.7)	6 (15.4)
South	114 (18.4)	36 (15.1)	23 (18.1)	7 (17.5)	9 (19.1)	3 (17.6)	24 (21.2)	12 (30.8)
West	107 (17.2)	39 (16.4)	18 (14.2)	10 (25.0)	6 (12.8)	6 (35.3)	20 (17.7)	8(20.5)
**Ethnic background**								
No migrant background (Dutch)	581 (93.6)	223 (93.7)	121 (95.3)	37 (92.5)	44 (93.6)	16 (94.1)	104 (92.0)	36 (92.3)
Migrant background	40 (6.4)	15 (6.3)	6 (4.7)	3 (7.5)	3 (6.4)	1 (5.9)	9 (8.0)	3 (7.7)
**Marital status**								
Single	12 (1.9)	1 (0.4)	4 (3.1)	2 (5.0)	0 (0.0)	1 (5.9)	3 (2.7)	1 (2.6)
Partner	609 (98.1)	237 (99.6)	123 (96.9)	38 (95.0)	47 (100.0)	16 (94.1)	110 (97.3)	38 (97.4)
**Religion**								
None	437 (70.4)	160 (67.2)	85 (66.9)	29 (72.5)	36 (76.6)	15 (88.2)	82 (72.6)	30 (76.9)
Christianity, Islam or other	184 (29.6)	78 (32.8)	42 (33.1)	11 (27.5)	11 (23.4)	2 (11.8)	31 (27.4)	9 (23.1)
**Education level**								
Low	47 (7.6)	13 (5.5)	8 (6.3)	6 (15.0)	2 (4.3)	0 (0.0)	15 (13.3)	3 (7.7)
Middle	268 (43.2)	96 (40.3)	66 (52.0)	13 (32.5)	22 (46.8)	6 (35.3)	54 (47.8)	11 (28.2)
High	306 (49.3)	129 (54.2	53 (41.7)	21 (52.5)	23 (48.9)	11 (64.7)	44 (38.9)	25 (64.1)
**Income**								
< €2000	109 (17.6)	42 (17.6)	20 (15.7)	7 (17.5)	2 (4.3)	6 (35.3)	27 (23.9)	5 (12.8)
€2000-€2500	41 (6.6)	18 (7.6)	8 (6.3)	2 (5.0)	4 (8.5)	0 (0.0)	8 (7.1)	1 (2.6)
€2501-€3500	199 (32.0)	73 (30.7)	40 (31.5)	8 (20.0)	18 (38.3)	3 (17.6)	41 (36.3)	16 (41.0)
> €3500	221 (35.6)	85 (35.7)	49 (38.6)	17 (42.5)	19 (40.4)	7 (41.2)	28 (24.8)	16 (41.0)
Unknown/does not want to answer	51 (8.2)	20 (8.4)	10 (7.9)	6 (15.0)	4 (8.5)	1 (5.9)	9 (8.0)	1 (2.6)

**Table 2:** Pregnancy, birth, birth interventions and healthcare professionals characteristics of the included Dutch women (*N* = 621)

	Total population	Community midwife	Hospital-based midwife	Obstetrician	Community midwife and Hospital-based midwife	Community midwife and Obstetrician	Hospital-based midwife and Obstetrician	Community midwife, Hospital-based midwife and Obstetrician
	***N*** = 621 100%	***n*** = 238 38.3%	***n*** = 127 20.5%	***n*** = 40 6.4%	***n*** = 47 7.6%	***n*** = 17 2.7%	***n*** = 113 18.2%	***n*** = 39 6.3%
	N (%)	n(%)	n(%)	n(%)	n(%)	n(%)	n(%)	n(%)
**PREGNANCY CHARACTERISTICS**								
**Parity**								
Nulliparous	289 (46.5)	76 (31.9)	48 (37.8)	23 (57.5)	29 (61.7)	10 (58.8)	38 (33.6	34 (87.2)
Multiparous	332 (53.5)	162 (68.1)	79 (62.2)	17 (42.5)	18 (38.3)	7 (41.2)	75 (66.4)	5 (12.8)
**Gestational age (in weeks)**								
≤ 36 + 6	19 (3.1)	0 (0.0)	7 (5.5)	5(12.5)	0 (0.0)	0 (0.0)	6 (5.3)	1 (2.6)
37 + 0 - 39 + 6	310 (49.9)	103 (43.3)	78 (61.4)	21 (52.5)	26 (55.3)	7 (41.2)	60 (53.1)	15 (38.5)
40 + 0 - 40 + 6	164 (26.4)	86 (36.1)	14 (11.0)	8 (20.0)	15 (31.9)	6 (35.3)	23 (20.4)	12 (30.8)
41 + 0 - 41 + 6	117 (18.8)	46 (19.3)	27 (21.3)	4 (10.0)	6 (12.8)	4 (23.5)	20 (17.7)	10 (25.6)
≥ 42 + 0	11 (1.8)	3 (1.3)	1 (0.8)	2 (5.0)	0 (0.0)	0 (0.0)	4 (3.5)	1 (2.6)
**Adverse physical health events** ^a^								
None	333 (53.6)	174 (73.1)	52 (40.9)	15 (37.5)	30 (63.8)	7 (41.2)	31 (27.4)	24 (61.5)
1	168 (27.1)	45 (18.9)	39 (30.7)	14 (35.0)	13 (27.7)	8 (47.1)	41 (36.3)	8 (20.5)
2	82 (13.2)	13 (5.5)	26 (20.5)	8 (20.)	3 (6.4)	2 (11.8)	26 (23.0)	4 (10.3)
≥ 3	38 (6.1)	6 (2.5)	10 (7.9)	3 (7.5)	1 (2.1)	0 (0.0)	15 (13.3)	3 (7.7)
**Adverse psychological health status** ^b^								
None	602 (96.9)	234 (98.3)	123 (96.9)	36 (90.0)	46 (97.9)	16 (94.1)	110 (97.3)	37 (94.9)
1	19 (3.1)	4 (1.7)	4 (3.1)	4 (10.0)	1 (2.1)	1 (5.9)	3 (2.7)	2 (5.1)
**BIRTH CHARACTERISTICS**								
**Mode of birth**								
Vaginal	509 (82.0)	238 (100.0)	127 (100.0)	18 (45.0)	47 (100.0)	9 (52.9)	53 (46.9)	17 (43.6)
Instrumental vaginal	45 (7.2)	0 (0.0)	0 (0.0)	3 (7.5)	0 (0.0)	5 (29.4)	20 (17.7)	17 (43.6)
CS (elective or emergency)	67 (10.8)	0 (0.0)	0 (0.0)	19 (47.5)	0 (0.0)	3 (17.6)	40 (35.4)	5 (12.8)
**Number of times the attended healthcare professional during birth was met in the prenatal period**								
0	214 (34.5)	20 (8.4)	96 (75.6)	14 (35.0)	15 (31.9)	0 (0.0)	60 (53.1)	9 (23.1)
1-2 times	110 (17.7)	48 (20.2)	10 (7.9)	13 (32.5)	6 (12.8)	1 (5.9)	22 (19.5)	10 (25.6)
3-4 times	113 (18.2)	74 (31.1)	7 (5.5)	3 (7.5)	10 (21.3)	5 (29.4)	7 (6.2)	7 (17.9)
≥ 5 times	150 (24.2)	82 (34.5)	11 (8.7)	6 (15.0)	14 (29.8)	11 (64.7)	16 (14.2)	10 (25.6)
Unknown	34 (5.5)	14 (5.9)	3 (2.4)	4 (10.0)	2 (4.3)	0 (0.0)	8 (7.1)	3 (7.7)
**Place of giving birth**								
Home	157 (25.3)	156 (65.5)	0 (0.0)	0 (0.0)	0 (0.0)	1 (5.9)	0 (0.0)	0 (0.0)
Maternity hotel/birth centre	29 (4.7)	9 (3.8)	6 (4.7)	2 (5.0)	1 (2.1)	2 (11.8)	7 (6.2)	2 (5.1)
Hospital	435 (70.0)	73 (30.7)	121 (95.3)	38 (95.0)	46 (97.9)	14 (82.4)	106 (93.8)	37 (94.9)
**BIRTH INTERVENTIONS**								
**Induction of labour**								
No	467 (75.2)	238 (100.0)	59 (46.5)	24 (60.0)	44 (93.6)	16 (94.1)	54 (47.8)	32 (82.1)
Yes	154 (24.8)	0 (0.0)	68 (53.5)	16 (40.0)	3 (6.4)	1 (5.9)	59 (52.2)	7 (17.9)
**CTG monitoring during labour**								
No	307 (49.4)	223 (97.9)	24 (18.9)	9 (22.5)	14 (29.8)	7 (41.2)	12 (10.6)	8 (20.5)
Yes	314 (50.6)	5 (2.1)	103 (81.1)	31 (77.5)	33 (70.2)	10 (58.8)	101 (89.4)	31 (79.5)
**Episiotomy** ^**c**^								
No	453 (81.8)	225 (94.5)	112 (88.2)	15 (71.4)	39 (83.0)	10 (71.4)	36 (49.3)	16 (47.1)
Yes	101 (18.2)	13 (5.5)	15 (11.8)	6 (28.6)	8 (17.0)	4 (28.6)	37 (50.7)	18 (52.9)
**Pain relief treatment** ^d^								
No	433 (69.7)	238 (100.0)	76 (59.8)	19 (47.5)	30 (63.8)	10 (58.8)	37 (32.7)	23 (59.0)
Yes	188 (30.3)	0 (0.0)	51 (40.2)	21 (52.5)	17 (36.2)	7 (41.2)	76 (67.3)	16 (41.0)
**Catheterisation**								
No	383 (61.7)	212 (89.1)	77 (60.6)	17 (42.5)	23 (48.9)	6 (35.3)	34 (30.1)	14 (35.9)
Yes	238 (38.3)	26 (10.9)	50 (39.4)	23 (57.5)	24 (51.1)	11 (64.7)	79 (69.9)	25 (64.1)
**Vaginal laceration** ^**c**^								
No	209 (37.7)	103 (43.3)	46 (36.2)	7 (33.3)	14 (29.8)	3 (21.4)	28 (38.4)	8 (23.5)
Yes	345 (62.3)	135 (56.7)	81 (63.8)	14 (66.7)	33 (70.2)	11 (78.6)	45 (61.6)	26 (76.5)
**Vaginal laceration OK treatment** ^**c**^								
No	526 (94.9)	228 (95.8)	124 (97.6)	20 (95.2)	45 (95.7)	13 (92.9)	67 (91.8)	29 (85.3)
Yes	28 (5.1)	10 (4.2)	3 (2.4)	1 (4.8)	2 (4.3)	1 (7.1)	6 (8.2)	5 (14.7)
**Manual placenta removal**								
No	597 (96.1)	233 (97.9)	124 (97.6)	39 (97.5)	44 (93.6)	17 (100.0)	105 (92.9)	35 (89.7)
Yes	24 (3.9)	5 (2.1)	3 (2.4)	1 (2.5)	3 (6.4)	0 (0.0)	8 (7.1)	4 (10.3)
**NEONATAL OUTCOMES**								
**Resuscitation**								
No	595 (95.8)	234 (98.3)	123 (96.9)	38 (95.0)	47 (100.0)	16 (94.1)	100 (88.5)	37 (94.9)
Yes	26 (4.2)	4 (1.7)	4 (3.1)	2 (5.0)	0 (0.0)	1 (5.9)	13 (11.5)	2 (5.1)

^a^ Adverse physical health events during pregnancy: intra-uterine growth restriction, blood loss trimester first trimester, blood loss second or third trimester, diabetes gravidarum, cholestasis, problems with blood pressure, abruptio placenta, placenta praevia, polyhydramnion, thrombosis, HELLP-syndrome, dysmature, macrosomia, oligohydramnion

^b^ Adverse psychological health status during pregnancy: anxiety or depression

^c^ Episiotomy, vaginal laceration and vaginal laceration which required OK-treatment were calculated for vaginal births only (*n* = 554)

^d^ Pain relief include epidural, morphine or pethidine treatment

In the included population a median MADM score of 35 (IQR 25-41) was observed, this score ranged from 7 to 42. The median score of the MORi was 75 (IQR 69-78), ranging from 35 to 84. The weighted mean CEQ2.0 was 3.25 (SD 0.53), ranging from 1 to 4.

### Reliability

Regarding the MADM, the Cronbach’s alpha ranged from 0.96-0.97, whereas for the MORi the range was 0.77-0.84. The CEQ2.0 showed a Cronbach’s alpha of 0.92, and the range for its four subscales was 0.78-0.85 (Table 3).

**Table 3:** Reliability analysis of the Dutch versions of the Mothers Autonomy in Decision Making Scale, Mothers on Respect Index and Childbirth Experience Questionnaire 2.0

	Completed measures	Number of items	Cronbach’s alpha
Mothers’ Autonomy in Decision Making scale			
Community midwife	341	7	0.97
Hospital-based midwife	326	7	0.96
Obstetrician	209	7	0.96
Mothers on Respect Index			
Community midwife	341	14	0.77
Hospital-based midwife	326	14	0.80
Obstetrician	209	14	0.84
Childbirth Experience Questionnaire			
Total	621	22	0.92
Domain: Own capacity	621	8	0.83
Domain: Perceived safety	621	6	0.85
Domain: Professional support	621	5	0.83
Domain: Participation	621	3	0.78

### Known group validity

Our analysis (Table 4) showed no statistical differences on MADM, MORi and CEQ2.0 scores between subgroups who differed on demographic characteristics, except for women who differed on monthly income and maternal age (Table 4). Women who differed on maternal age, showed that women in the age group ≥36 years had the highest CEQ2.0-scores compared with the other age groups. Women with a lower income showed statistically significantly lower scores on the MORi.

**Table 4:** Scores on the Mothers Autonomy in Decision Making Scale, Mothers on Respect Index and Childbirth Experience Questionnaire 2.0 of the total population who differed on maternal characteristics (*N* = 621)

	Mothers’ Autonomy in Decision Making scale ^a^	Statistical differences among subgroups total population	Mothers on Respect Index ^a^	Statistical differences among subgroups total population	Childbirth Experience Questionnaire 2.0	Statistical differences among subgroups total population
	Median (IQR)	***p***-value	Median (IQR)	***p***-value	Mean (SD)	***p***-value
**MATERNAL CHARACTERISTICS**						
**Maternal age (in years)**		0.11		0.06		**0.05**
≤ 25	35 (24-41)		74 (68-78)		3.17 (0.60)	
26-30	35 (25-41)		75 (69-79)		3.28 (0.51)	
31-35	34 (25-40)		74 (68-78)		3.21 (0.55)	
≥ 36	36 (28-42)		76 (72-79)		3.38 (0.51)	
**Dutch regions**		0.89		0.92		0.69
North	35 (26-40)		74 (68-79)		3.25 (0.58)	
East	35 (24-41)		75 (71-78)		3.31 (0.48)	
South	35 (25-42)		75 (69-79)		3.24 (0.54)	
West	35 (25-40)		74 (70-79)		3.25 (0.50)	
**Ethnic background**		0.53		0.91		0.25
No migrant background (Dutch)	35 (26-41)		75 (69-78)		3.27 (0.53)	
Migrant background	34 (24-42)		74 (69-79)		3.14 (0.66)	
**Marital status**		0.87		0.63		0.18
Single	34 (21-42)		73 (62-79)		3.26 (0.53)	
Partner	35 (25-41)		75 (69-78)		3.05 (0.63)	
**Religion**		0.28		0.84		0.28
None	35 (26-41)		75 (69-79)		3.25 (0.55)	
Christianity, Islam or other	35 (23-41)		74 (70-78)		3.30 (0.51)	
**Education level**		0.49		0.15		0.09
Low	35 (24-41)		74 (64-78)		3.12 (0.65)	
Middle	35 (23-41)		74 (69-78)		3.25 (0.57)	
High	35 (27-41)		75 (70-79)		3.30 (0.48)	
**Income**		0.82		**0.03**		0.07
< €2000	35 (24-40)		74 (66-78)		3.20 (0.60)	
€2000-€2500	34 (27-41)		74 (71-79)		3.21 (0.46)	
€2501-€3500	35 (24-41)		74 (69-78)		3.21 (0.57)	
> €3500	35 (26-40)		75 (70-79)		3.31 (0.48)	
Unknown/does not want to answer	35 (29-42)		77 (73-79)		3.40 (0.48)	

^a^ MADM scores and MORi scores were at random selected if a women had completed these for each multiple healthcare professional that they encountered for in the intrapartum period

Compared with women who experienced a healthy pregnancy and had a physiological birth, women who had pregnancy complications, or who experienced birth interventions, had statistically significantly lower scores on the MADM, MORi and CEQ2.0. No statistical significant differences on MADM, MORi and CEQ2.0 scores were observed for women who differed in vaginal laceration repair either with or without surgery (Tables 5, 6, 7). We did not calculate any statistical differences for adverse neonatal outcomes, since none of the women reported newborn/child mortality, and the prevalence of resuscitation was rather low (4.2%).

**Table 5:** Median (interquartile range) scores on the Mothers Autonomy in Decision Making Scale for the total population and stratified for maternal healthcare providers who provided care during labour and birth, 621 Dutch women completed 876 measures

	Total population^a^	Statistical differences among subgroups total population	Community midwife	Hospital-based midwife	Obstetrician	Community midwife and hospital-based midwife	Community midwife and Obstetrician	Hospital-based midwife and Obstetrician	Community midwife, Hospital-based midwife and Obstetrician
	*N* = 621 (100%)		*n* = 238 (38.3%)	*n* = 127 (20.5%)	*n* = 40 (6.4%)	*n* = 47 (7.6%)	*n* = 17 (2.7%)	*n* = 113 (18.2%)	*n* = 39 (6.3%)
	Median (IQR)	*P*-value	Median (IQR)	Median (IQR)	Median (IQR)	Median (IQR)	Median (IQR)	Median (IQR)	Median (IQR)
**PREGNANCY CHARACTERISTICS**									
**Parity**		**0.01**							
Nulliparous	34 (23-40)		38 (34-42)	30 (15-38)	29 (17-36)	39 (34-42) 29 (23-35)	35 (31-39) 27 (19-35)	31 (23-35) 29 (16-35)	35 (31-42) 26 (16-36) 25 (14-35)
Multiparous	35 (28-41)		39 (35-42)	32 (25-35)	35 (23-39)	37 (35-41) 20 (16-34)	36 (34-39) 23 (13-34)	33 (21-37) 32 (24-35)	34 (17-41) 14 (11-42) 14 (11-32)
**Gestational age (in weeks)**		**0.05**							
≤ 36 + 6	27 (23-35)		NA	27 (25-34)	23 (7-27)	NA	NA	31 (22-37) 35 (23-42)	42 NA 42 NA 42 NA
37 + 0 – 39 + 6	35 (25-41)		39 (35-42)	31 (19-36)	36 (33-42)	39 (34-42) 30 (14-37)	35 (29-36) 27 (13-35)	32 (19-36) 29 (15-36)	35 (27-42) 28 (16-35) 25 (14-35)
40 + 0 – 40 + 6	35 (28-41)		38 (33-42)	34 (29-35)	29 (21-36)	38 (34-41) 29 (20-33)	34 (31-38) 23 (9-28)	32 (23-35) 28 (21-35)	35 (28-41) 26 (14-42) 24 (11-39)
41 + 0 – 41 + 6	35 (24-41)		40 (35-42)	32 (23-40)	22 (12-31)	39 (27-42) 22 (16-34)	39 (37-39) 31 (24-40)	35 (26-35) 33 (25-35)	37 (30-42) 22 (13-38) 15 (12-35)
≥ 42 + 0	26 (20-35)		35 NA	42 NA	23 NA	NA	NA	19 (9-26) 26 (15-35)	29 NA 25 NA 24 NA
**Complications during pregnancy** ^b^		**0.01**							
None	35 (27-41)		39 (34-42)	31 (18-36)	35 (23-36)	40 (35-42) 29 (17-33)	36 (29-39) 25 (21-27)	32 (24-35) 34 (21-37)	35 (28-42) 26 (15-38) 23 (14-35)
1	35 (25-41)		41 (35-42)	32 (27-35)	31 (14-38)	36 (34-42) 29 (18-36)	35 (32-38) 29 (10-35)	32 (23-36) 30 (16-35)	39 (34-42) 21 (14-37) 21 (8-34)
2	35 (25-37)		38 (35-42)	33 (26-38)	29 (21-39)	34 NA 34 NA	35 NA 28 NA	32 (16-35) 33 (16-36)	36 (28-41) 33 (24-41) 36 (19-41)
≥ 3	27 (18-36)		34 (22-42)	27 (16-37)	23 NA	20 NA 17 NA	NA	31 (19-35) 27 (17-35)	34 (NA) 25 (NA) 19 (NA)
**BIRTH CHARACTERISTICS**									
**Mode of birth**		**≤ 0.001**							
Vaginal	35 (27-41)		39 (34-42)	32 (21-36)	31 (19-37)	38 (34-42) 29 (17-35)	36 (34-40) 27 (11-35)	31 (15-35) 27 (14-36)	38 (34-42) 28 (14-39) 26 (14-42)
Instrumental vaginal	29 (17-35)		NA	NA	31 NA	NA	34 (31-35) 22 (14-33)	31 (26-35) 24 (16-33)	35 (31-42) 26 (16-39) 16 (12-34)
CS (elective or emergency)	32 (25-36)		NA	NA	35 (23-39)	NA	39 NA 27 NA	34 (26-36) 35 (27-39)	27 (10-31) 23 (9-31) 25 (16-32)
**Number of times the attended healthcare professional during birth was met in the prenatal period**		**≤ 0.001**							
0	32 (21-37)		38 (25-42)	31 (20-35)	32 (22-36)	37 (33-42) 29 (17-41)	NA	32 (23-36) 29 (15-35)	40 (33-42) 35 (30-40) 14 (25-39)
1-2 times	35 (22-42)		39 (31-42)	39 (29-42)	31 (22-40)	41 (37-42) 27 (19-35)	34 NA 20 NA	28 (16-34) 31 (12-35)	35 (27-40) 26 (15-36) 31 (10-38)
3-4 times	36 (32-41)		38 (34-42)	32 (26-42)	31 NA	36 (31-41) 29 (21-36)	36 (32-38) 23 (14-30)	36 (32-38) 36 (34-42)	35 (25-42) 25 (11-34) 19 (8-35)
≥ 5 times	36 (30-42)		40 (35-42)	34 (19-41)	37 (33-41)	39 (28-42) 26 (13-33)	35 (32-39) 27 (13-35)	35 (19-36) 35 (22-38)	36 (28-41) 18 (14-39) 20 (9-27)
Unknown	31 (21-39)		37 (29-42)	34 NA	7 (7-25)	38 NA 29 NA	NA	30 (25-33) 27 (23-29)	42 NA 42 NA 42 NA
**Place of giving birth**		**≤ 0.001**							
Home	39 (35-42)		39 (35-42)						
Maternity hotel/birth centre	34 (25-35)		34 (32-39)	32 (21-36)	36 NA	39 NA 39 NA	34 NA 33 NA	26 (25-34) 24 (14-35)	33 NA 30 NA 30 NA
Hospital	34 (24-39)		39 (34-42)	30 (12-37)	31 (22-37)	38 (34-42) 29 (17-34)	36 (31-39) 24 (12-34)	32 (22-35) 32 (17-35)	35 (29-42) 26 (14-38) 22 (14-35)
**BIRTH INTERVENTIONS**									
**Induction of labour**		**≤ 0.001**							
No	35 (27-41)		39 (34-42)	30 (21-35)	35 (23-36)	39 (34-42) 29 (18-35)	36 (32-39) 26 (15-33)	31 (22-35) 27 (18-36)	35 (28-42) 25 (14-37) 19 (11-35)
Yes	32 (20-37)		NA	33 (20-39)	31 (16-40)	20 NA 17 NA	NA	32 (23-35) 34 (17-35)	40 (29-42) 33 (25-38) 35 (25-37)
**CTG monitoring during labour**		**≤ 0.001**							
No	37 (32-42)		39 (35-42)	34 (29-38)	24 (13-36)	42 (30-42) 30 (19-36)	36 (34-39) 25 (21-35)	29 (10-33) 24 (9-35)	35 (28-35) 15 (13-33) 14 (8-15)
Yes	32 (22-36)		35 (21-37)	31 (19-36)	31 (23-38)	37 (34-41) 28 (17-34)	35 (31-39) 27 (12-34)	32 (24-35) 32 (18-35)	37 (29-42) 28 (17-38) 26 (16-35)
**Episiotomy** ^**c**^		**≤ 0.001**							
No	35 (29-42)		39 (34-42)	32 (20-36)	29 (16-37)	39 (34-42)	39 (34-42) 29 (16-37)	32 (20-36) 29 (16-37)	39 (34-42) 32 (30-36) 29 (16-37)
Yes	30 (16-36)		35 (31-40)	26 (17-35)	22 (14-35)	35 (31-40)	35 (31-40) 22 (14-35)	26 (17-35) 22 (14-35)	35 (31-40) 26 (17-35) 22 (14-35)
**Pain relief treatment** ^**d**^		**0.002**							
No	35 (27-41)		39 (34-42)	31 (21-35)	31 (23-36)	40 (35-42) 26 (17-35)	36 (32-37) 26 (12-34)	27 (19-35) 26 (14-35)	37 (31-42) 33 (14-42) 25 (10-35)
Yes	32 (22-39)		NA	35 (19-42)	34 (17-42)	35 (26-42) 31 (17-35)	35 (32-39) 27 (22-35)	33 (24-36) 33 (21-38)	35 (27-40) 26 (17-35) 22 (14-35)
**Catheterisation**		**≤ 0.001**							
No	36 (29-42)		39 (35-42)	32 (22-38)	30 (15-36)	41 (35-42) 26 (16-35)	37 (33-41) 22 (8-29)	31 (20-35) 26 (14-35)	39 (33-42) 35 (14-42) 28 (9-42)
Yes	32 (22-37)		39 (34-42)	31 (17-35)	34 (25-41)	35 (32-41) 30 (21-35)	35 (32-36) 27 (22-35)	32 (25-35) 32 (21-36)	35 (27-40) 25 (15-35) 22 (14-34)
**Vaginal laceration** ^**c**^		0.28							
No	35 (28-41)		39 (34-42)	31 (19-35)	29 (14-35)	39 (34-42)	39 (34-42) 29 (14-35)	31 (19-35) 29 (14-35)	39 (34-42) 31 (19-35) 29 (14-35)
Yes	35 (24-41)		38 (34-42)	31 (19-35)	26 (14-35)	38 (34-42)	38 (34-42) 26 (14-35)	31 (19-35) 26 (14-35)	38 (34-42) 31 (19-35) 26 (14-35)
**Vaginal laceration (surgery)** ^**c**^		0.48							
No	35 (25-41)		39 (34-42)	31 (19-35)	26 (14-35)	39 (34-42)	39 (34-42) 26 (14-35)	29 (19-35) 26 (14-35)	39 (34-42) 31 (19-35) 26 (14-35)
Yes	34 (25-38)		35 (31-39)	29 (15-35)	29 (14-35)	35 (31-39)	35 (31-39) 29 (13-35)	29 (15-35) 29 (13-35)	35 (31-39) 29 (13-35) 29 (13-35)

^a^ Total population *N* = 621, scores of the Mothers Autonomy in Decision Making Scale were at random selected if a women had multiple healthcare professionals

^b^ Complications during pregnancy care: intra-uterine growth restriction, blood loss trimester first trimester, blood loss second or third trimester, diabetes gravidarum, cholestasis, problems with blood pressure, abruptio placenta, placenta praevia, polyhydramnion, thrombosis, HELLP-syndrome, dysmature, macrosomia, oligohydramnion

^c^ Scores on the Mothers Autonomy in Decision Making Scale for birth interventions episiotomy, vaginal laceration and vaginal laceration which required OK-treatment were calculated for vaginal births only (*n* = 554)

^d^ Pain relief include epidural, morphine or pethidine treatment

*NA* Not Applicable

**Table 6:** Median (interquartile range) scores of the Mothers on Respect Index (interquartile range) scores for the total population and stratified for maternal healthcare providers who provided care during labour and birth, 621 Dutch women completed 876 MORi measures

	Total population ^**a**^	Statistical differences among subgroups total population	Community midwife	Hospital-based Midwife	Obstetrician	Community midwife and Hospital-based midwife	Community midwife and Obstetrician	Hospital-based midwife and Obstetrician	Community midwife, Hospital-based midwife and Obstetrician
	***N*** = 621 (100%)		***n*** = 238 (38.3%)	***n*** = 127 (20.5%)	***n*** = 40 (6.4%)	***n*** = 47 (7.6%)	***n*** = 17 (2.7%)	***n*** = 113 (18.2%)	***n*** = 39 (6.3%)
	Median (IQR)	***P***-value	Median (IQR)	Median (IQR)	Median (IQR)	Median (IQR)	Median (IQR)	Median (IQR)	Median (IQR)
**PREGNANCY CHARACTERISTICS**									
**Parity**		**0.01**							
Nulliparous	74 (68-78)		79 (74-79)	72 (67-78)	71 (64-75)	75 (72-79) 71 (68-76)	74 (69-78) 75 (70-78)	69 (62-73) 74 (65-78)	77 (69-79) 69 (59-75) 75 (63-79)
Multiparous	76 (71-79)		78 (74-79)	70 (59-76)	75 (62-79)	75 (73-79) 72 (59-75)	79 (74-79) 66 (46-72)	69 (62-74) 71 (67-77)	77 (77-82) 75 (63-78) 79 (65-84)
**Gestational age (in weeks)**		**0.02**							
< 36 + 6	70 (64-75)			70 (64-74)	70 (54-75)	NA	NA	71 (68-78) 77 (71-80)	64 NA 58 NA 49 NA
37 + 0 – 39 + 6	75 (69-78)		78 (74-79)	72 (63-78)	75 (66-79)	77 (73-79) 72 (68-77)	75 (69-79) 76 (70-79)	69 (60-74) 70 (64-79)	77 (71-79) 72 (67-77) 77 (63-79)
40 + 0 – 40 + 6	75 (71-79)		78 (74-79)	72 (69-77)	69 (61-75)	74 (72-78) 70 (64-72)	74 (69-80) 69 (60-72)	69 (66-72) 72 (67-77)	78 (73-79) 67 (61-78) 79 (71-82)
41 + 0 – 41 + 6	75 (69-78)		78 (75-79)	69 (64-78)	61 (45-73)	74 (71-79) 69 (66-72)	78 (76-79) 73 (68-78)	73 (67-74) 74 (68-77)	78 (73-80) 68 (58-75) 74 (62-76)
≥ 42 + 0	71 (58-76)		76 NA	78 NA	72 NA	NA	NA	64 (57-70) 65 (50-76)	56 NA 53 NA 61 NA
**Complications during pregnancy** ^a^		**0.01**							
None	76 (71-79)		78 (74-79)	72 (65-76)	74 (68-78)	77 (74-79) 72 (67-76)	79 (70-79) 70 (66-79)	69 (65-74) 75 (69-77)	78 (69-79) 68 (59-77) 74 (64-79)
1	74 (67-78)		78 (74-79)	73 (64-77)	70 (55-75)	74 (71-79) 71 (69-73)	75 (72-79) 75 (67-77)	69 (65-74) 74 (66-78)	75 (66-78) 72 (52-76) 77 (75-79)
2	74 (66-78)		76 (74-79)	74 (65-78)	70 (61-78)	71 NA 68 NA	72 NA 72 NA	70 (60-74) 74 (66-78)	76 (74-79) 76 (69-78) 76 (65-79)
≥ 3	72 (65-76)		NA	72 (50-77)	75 NA	45 NA 41 NA	NA	70 (61-72) 70 (64-78)	77 NA 66 NA 60 NA
**BIRTH CHARACTERISTICS**									
**Mode of birth**		**≤ 0.001**							
Vaginal	75 (70-79)		78 (74-79)	72 (64-78)	71 (64-76)	75 (72-79) 72 (68-75)	75 (74-78) 72 (57-77)	69 (62-74) 73 (66-78)	78 (75-79) 72 (63-77) 75 (65-79)
Instrumental vaginal	70 (66-78)		NA	NA	70 NA	NA	71 (69-79) 71 (67-78)	68 (63-72) 69 (66-76)	77 (70-79) 72 (58-78) 74 (63-79)
CS (elective or emergency)	73 (65-77)		NA	NA	74 (60-79)	NA	79 NA 70 NA	70 (62-74) 74 (66-78)	68 (56-75) 63 (50-70) 75 (67-77)
**Place of giving birth**		**≤ 0.001**							
Home	78 (74-79)		78 (74-79)	NA	NA	NA	79 NA 79 NA	V	NA
Hospital	73 (67-78)		78 (74-79)	72 (65-78)	73 (63-76)	75 (72-79) 72 (68-75)	75 (70-79) 71 (66-75)	69 (62-74) 73 (66-77)	77 (72-79) 70 (61-76) 75 (64-79)
Maternity hotel/birth centre	75 (68-78		76 (73-79)	64 (49-73)	73 NA	78 NA 76 NA	74 NA 77 NA	68 (60-75) 78 (68-79)	68 NA 66 NA 70 NA
**Number of times the attended healthcare professional during birth was met in the prenatal period**		**≤ 0.001**							
0	73 (66-78)		76 (74-79)	72 (64-78)	72 (62-77)	75 (71-79) 71 (68-77)	NA	71 (63-74) 74 (69-77)	77 (77-80) 72 (70-78) 76 (63-79)
1-2 times	74 (70-78)		78 (74-79)	76 (69-78)	73 (63-78)	76 (73-78) 70 (65-73)	67 NA 71 NA	69 (55-73) 72 (66-77)	77 (72-79) 65 (60-76) 76 (70-79)
3-4 times	76 (72-79)		78 (74-79)	76 (71-78)	70 NA	75 (72-78) 72 (68-76)	77 (72-79) 70 (65-72)	71 (62-74) 76 (65-79)	73 (69-79) 68 (63-72) 74 (61-77)
≥ 5	77 (72-79)		79 (76-79)	69 (64-73)	76 (74-79)	77 (73-79) 73 (60-77)	75 (71-79) 76 (67-79)	69 (55-76) 68 (63-77)	76 (67-79) 65 (54-75) 75 (59-79)
Unknown	70 (65-78)		78 (69 -79)	65 NA	56 (43-74)	75 NA 71 NA	NA	66 (62-72) 68 (66-74)	77 NA 75 NA 79 NA
**BIRTH INTERVENTIONS**									
**Induction of labour**		**≤ 0.001**							
No	75 (70-79)		78 (74-79)	71 (64-76)	74 (63-77)	75 (73-79) 72 (68-75)	75 (70-79) 71 (66-76)	69 (64-74) 72 (66-77)	77 (70-79) 68 (59-77) 74 (63-79)
Yes	72 (66-78)		NA	74 (66-78)	72 (64-76)	70NA 74 NA	79 NA 79 NA	69 (61-74) 74 (66-77)	77 (73-80) 72 (67-75) 77 (69-79)
**CTG during labour**		**≤ 0.001**							
No	77 (73-79)		78 (74-79)	71 (64-78)	64 (50-77)	78 (73-79) 72 (68-78)	77 (74-79) 71 (66-77)	67 (61-73) 70 (64-75)	77 (74-78) 68 (62-72) 73 (62-79)
Yes	72 (66-77)		78 (67-79)	72 (65-77)	73 (68-76)	75 (71-78) 70 (63-75)	74 (69-79) 72 (62-77)	70 (62-74) 74 (67-77)	77 (68-77) 77 (72-58) 75 (65-79)
**Episiotomy** ^**c**^		**≤ 0.001**							
No	76 (71-79)		78 (74-79)	72 (65-77)	73 (67-79)	78 (74-79) 72 (65-76)	78 (74-79) 73 (67-79)	72 (65-77) 73 (67-79)	78 (74-79) 72 (65-77) 73 (67-79)
Yes	71 (67-77)		76 (73-79)	69 (62-72)	71 (63-77)	77 (74-79) 71 (62-76)	76 (73-79) 71 (63-77)	69 (62-72) 71 (63-77)	76 (73-79) 69 (69-73) 71 (63-77)
**Pain relief treatment** ^**d**^		**≤ 0.001**							
No	76 (70-79)		78 (74-79)	72 (62-76)	70 (64-75)	77 (74-79) 72 (68-74)	75 (71-79) 72 (60-76)	69 (62-73) 71 (67-78)	77 (72-79) 72 (60-78) 75 (69-79)
Yes	72 (66-78)		NA	72 (67-78)	75 (60-78)	74 (70-77) 71 (66-76)	77 (67-79) 71 (67-79)	71 (62-74) 74 (65-77)	77 (68-79) 67 (59-75) 74 (62-79)
**Catheterisation**		**≤ 0.001**							
No	76 (71-79)		78 (74-79)	73 (64-78)	68 (58-76)	77 (74-79) 72 (64-76)	79 (75-80) 68 (60-78)	69 (66-74) 71 (66-77)	78 (70-79) 72 (62-78) 77 (69-79)
Yes	68 (72-77)		76 (73-78)	71 (66-77)	75 (77-69)	75 (71-79) 71 (68-74)	74 (69-77) 72 (70-76)	70 (62-74) 74 (66-77)	77 (71-79) 68 (59-76) 74 (63-79)
**Vaginal laceration** ^**b**^		0.06							
No	76 (71-79)		78 (74-79)	72 (65-76)	70 (63-76)	74 (71-78) 72 (68-76)	78 (74-79) 70 (63-76)	72 (65-76) 70 (63-76)	78 (74-79) 72 (65-76) 70 (63-76)
Yes	75 (69-78)		77 (74-79)	71 (62-76)	74 (67-79)	76 (74-79) 71 (66-76)	77 (74-79) 74 (67-79)	71 (62-76) 74 (67-79)	77 (74-79) 71 (62-76) 74 (67-79)
**Vaginal laceration (surgery)** ^**b**^		0.73							
No	75 (69-79)		78 (74-79)	72 (64-76)	72 (64-77)	78 (74-79) 64 (72-76)	78 (74-79) 72 (64-77)	72 (64-76) 72 (64-77)	78 (74-79) 72 (64-76) 72 (64-77)
Yes	76 (70-79)		77 (72-79)	71 (65-75)	75 (68-79)	77 (72-79) 71 (65-75)	77 (72-79) 75 (68-79)	71 (65-75) 75 (68-79)	77 (72-79) 71 (65-75) 75 (68-79)

^a^ Total population *N* = 621, scores on the Mothers on Respect Index were at random selected if a women had multiple healthcare professionals

^b^ Complications during pregnancy care: intra-uterine growth restriction, blood loss trimester first trimester, blood loss second or third trimester, diabetes gravidarum, cholestasis, problems with blood pressure, abruptio placenta, placenta praevia, polyhydramnion, thrombosis, HELLP-syndrome, dysmature, macrosomia, oligohydramnion

^c^ Scores on the Mothers on Respect Index for birth interventions episiotomy, vaginal laceration and vaginal laceration which required OK-treatment were calculated for vaginal births only (*n* = 554)

^d^ Pain relief include epidural, morphine or pethidine treatment

*NA* Not Applicable

**Table 7:** Weighted mean (standard deviation) scores of the Childbirth Experience Questionnaire 2.0 for the total population (*n* = 621)

	Total population ***N*** = 621	Statistical differences among subgroups
	Mean (SD)	***P***-value
**PREGNANCY CHARACTERISTICS**		
**Parity**		**≤ 0.001**
Nulliparous	3.35 (0.50)	
Multiparous	3.16 (0.55)	
**Gestational age**		0.27
< 36 + 6	3.13 (0.48)	
37 + 0 - 39 + 6	3.24 (0.58)	
40 + 0 - 40 + 6	3.31 (0.49)	
41 + 0 - 41 + 6	3.28 (0.45)	
≥ 42 + 0	3.03 (0.58)	
**Complications during pregnancy** ^a^		**≤ 0.001**
None	3.36 (0.48)	
1	3.2 (0.51)	
2	3.13 (0.60)	
≥ 3	2.91 (0.66)	
**BIRTH CHARACTERISTICS**		
**Mode of birth**		**≤ 0.001**
Vaginal	3.32 (0.51)	
Instrumental vaginal	2.96 (0.44)	
CS (elective or emergency)	2.98 (0.62)	
**Number of times the attended healthcare professional during birth was met in the prenatal period**		**≤ 0.001**
0	3.13 (0.57)	
1-2	3.25 (0.54)	
3-4	3.44 (0.39)	
≥ 5	3.37 (0.50)	
Unknown	3.05 (0.59)	
**Place of giving birth**		**≤ 0.001**
Home	3.55 (0.37)	
Maternity hotel/birth centre	3.17 (0.54)	
Hospital	3.03 (0.60)	
**Healthcare professionals**		**≤ 0.001**
Community midwife	3.54 (0.36)	
Hospital-based midwife	3.17 (0.52)	
Obstetrician	3.02 (0.63)	
Community midwife and Hospital-based midwife	3.22 (0.49)	
Community midwife and Obstetrician	3.13 (0.43)	
Hospital-based midwife and Obstetrician	2.97 (0.58)	
Community midwife and Hospital-based midwife and Obstetrician	3.07 (0.55)	
**BIRTH INTERVENTIONS**		
**Induction of labour**		**≤ 0.001**
No	3.34 (0.49)	
Yes	3.03 (0.60)	
**CTG monitoring**		**≤ 0.001**
No	3.45 (0.43)	
Yes	3.08 (0.56)	
**Episiotomy** ^**b**^		**≤ 0.001**
No	3.31 (0.51)	
Yes	2.99 (0.59)	
**Pain relief treatment** ^**c**^		**≤ 0.001**
No	3.36 (0.49)	
Yes	3.04 (0.57)	
**Catheterisation**		**≤ 0.001**
No	3.37 (0.49)	
Yes	3.09 (0.56)	
**Vaginal laceration** ^**b**^		0.83
No	3.27 (0.55)	
Yes	3.26 (0.57)	
**Vaginal laceration (surgery)** ^**b**^		0.19
No	3.27 (0.53)	
Yes	3.16 (0.61)	

^a^ Complications during pregnancy care: intra-uterine growth restriction, blood loss trimester first trimester, blood loss second or third trimester, diabetes gravidarum, cholestasis, problems with blood pressure, abruptio placenta, placenta praevia, polyhydramnion, thrombosis, HELLP-syndrome, dysmature, macrosomia, oligohydramnios

^b^ Scores on the Childbirth Experience Questionnaire 2.0 for birth interventions episiotomy, vaginal laceration and vaginal laceration which required OK-treatment were calculated for vaginal births only (*n* = 554)

^c^ Pain relief include epidural, morphine or pethidine treatment

Women, who had two or three maternal health care providers attending at birth, gave community care midwives statistical significant higher scores on the MADM and MORi compared with a clinical midwife or obstetrician (Supplementary Table). This pattern of decreased MADM and MORi scores was observed for each pregnancy and birth characteristic. For example, major differences in MADM median scores were observed when care was provided by three maternal healthcare providers of which none was familiar with the women, community midwives scored 40 (IQR 33-42), hospital-based midwife 35 (IQR 30-40), and obstetrician 14 (IQR 25-39), respectively (Tables 5, 6 and 7).

### Convergent validity

The calculated Spearman Rank correlations between the separate measures of the MADM, MORi and CEQ2.0 ranged, as hypothesized, from 0.49-0.63 (Table 8).

**Table 8:** Convergent validity assessed with Spearman Rank correlations between the Mothers Autonomy in Decision Making Scale, Mothers on Respect Index and Childbirth Experience Questionnaire 2.0

	Mothers Autonomy in Decision Scale	Mothers on Respect Index
	Spearman Rank Correlation	Spearman Rank Correlation
Mothers Autonomy in Decision Scale	NA	0.57
Mothers on Respect Index	0.57	NA
Childbirth Experience Questionnaire 2.0	0.49	0.63

### Acceptability and clarity

Regarding the acceptability of completing the MADM and MORi for each healthcare provider that attended during the intrapartum period 639 (97.6%) women out of the eligible population of 655, who completed the online survey, had filled in the measures multiple times (Fig. 1). Regarding the clarity of the seven items of the MADM, 7 women (10.8%) indicated problems for completing item 1 “My midwife/obstetrician asked me how involved in decision making I wanted to be” and none had problems understanding item 4 “My midwife/obstetrician helped me understand all the information”. Regarding the clarity of the fourteen items of the MORi, women indicated the most problems with completing item 5 “I chose the care options that I received” (2.7%), item 2 “Comfortable declining care that was offered”(2.4%), and item 4 “Coerced into accepting the options my (midwife, doctor) suggested” (2.1%, Table 9).

**Table 9:** Women indicated difficulties in completion on the separate items of the Mothers Autonomy in Decision Making Scale and Mothers on Respect Index (*n* = 621)

	Difficult to complete item N (%)
Mothers’ Autonomy in Decision Making scale	
Item 1 My midwife/obstetrician asked me how involved in decision making I wanted to be	67 (10.8)
Item 2 My midwife/obstetrician told me there are different options for maternity care	22 (3.5)
Item 3 My midwife/obstetrician explained the advantages and disadvantages of the maternity care options	4 (0.6)
Item 4 My midwife/obstetrician helped me understand all information	0 (0.0)
Item 5 I was given enough time to thoroughly consider the different maternity care options	13 (2.1)
Item 6 I was able to choose what I consider to be the best car options	8 (1.3)
Item 7 My midwife/obstetrician respected that choice	4 (0.6)
Mothers on Respect Index	
Item 1 Comfortable asking questions	9 (1.4)
Item 2 Comfortable declining care that was offered	15 (2.4)
Item 3 Comfortable accepting the options for care that my midwife/obstetrician recommended	11 (1.8)
Item 4 Coerced into accepting the options my midwife/obstetrician suggested	13 (2.1)
Item 5 I chose the care options that I received	17 (2.7)
Item 6 My personal preferences were respected	10 (1.6)
Item 7 My personal cultural preferences were respected	
During childbirth I held back from asking questions of discussing my concerns	4 (0.6)
Item 8 Because my midwife/obstetrician seemed rushed	5 (0.8)
Item 9 Because I wanted maternity care differed from what my midwife/obstetrician recommended	4 (0.6)
Item 10 Because I thought my maternity care provider might think you were being difficult	4 (0.6)
During childbirth I felt that I was treated poorly by my midwife/obstetrician	
Item 11 Because of my race ethnicity, cultural background or language	6 (1.0)
Item 12 Because of my sexual orientation and/or gender identity	11 (1.8)
Item 13 Because of my supplementary health insurance	9 (1.4)
Item 14 Because of a difference in opinion with your caregivers about the right care for yourself of your baby	11 (1.8)

Additionally, women provided some general remarks on the overall completion of the MADM and MORi, they questioned if all items were applicable to complete since they had experienced a precipitous labour or an elective caesarean section.

## Discussion

The present study aimed to evaluate the psychometric qualities of three measures, the MADM, MORi and CEQ2.0, that assessed Dutch women’s experiences (*n* = 621) in the intrapartum period. All three instruments displayed good psychometric properties when used to assess experiences of intrapartum care. The reliability of the measures was good, and the calculated Cronbach’s alphas for the MADM, MORi and CEQ2.0, were ≥ 0.77. Women who differed on demographic characteristics showed similar scores on the MADM, MORi and CEQ2.0, except for those who differed in age and income. The three measures showed statistically significant different scores by variations in individual care characteristics (i.e. birth, birth interventions), except for vaginal laceration repair (knowngroup validity) and the convergent validity showed good to moderate correlations between the three measures. MADM and MORi, both measures had a promising uptake and completion even when multiple healthcare providers were encountered during the intrapartum period.

The reliability and construct validity of the MADM, MORi and CEQ2.0 showed similar results with previous studies in which psychometric evaluations were done [12, 19–23]. Based on our previous study we recommended completion of the MADM and MORi for each healthcare provider separately since in the Dutch healthcare system women can receive care from multiple maternal healthcare providers [12]. Our study showed that women were able and willing to complete both MADM and MORi for each healthcare provider they encountered during the intrapartum period. Regarding clarity, some women indicated that some items of the MADM and MORi were difficult to complete, since they experienced a precipitous labour or an elective caesarean section. Also, they indicated that they needed more clarity on the operationalization of their involvement in decision making in the intrapartum period. Preferably, starting in prenatal care, maternal healthcare providers should engage women in an anticipatory informed decision making process around their preferences during labour and birth and discuss how they can be actively involved in the process of decision making in the intrapartum period [40].

Women completed the MADM and MORi separately for each healthcare provider, showing a decline in these scores for each additional healthcare provider that cared for them in the intrapartum period. This finding is in line with previously published results, showing that women who received care from their community midwife showed statistically significantly higher scores on the MADM, MORi as well as CEQ2.0 [12, 19–23].The decline in scores could be explained due to unexpected complications that arised during labour and birth that require assistance of additional healthcare providers. This could have meant that when unexpected birth interventions occurred, providers attended to effecting the intervention without pausing to involve them in a detailed informed choice discussion, at the expense of their feeling of autonomy and control. When Dutch women are referred during childbirth from midwife-led care to obstetrician-led care, it is known that they experience less continuity of care, since they will receive care from a new team of maternal healthcare professionals (e.g. hospital based-midwife and/or obstetrician) [36]. These findings are substantiated by results from Australia, where women who received fragmented care during childbirth experienced lower autonomy, lower respect, and experienced lower childbirth experience compared to those who had continuity of care provided by a midwife or obstetrician [35].These findings corroborate the importance of providing continuity of care and the need for extra attention to those who need to be referred during childbirth.

### Strengths and limitations

The first strength is that in our sample, women completed the measures MADM and MORi for each healthcare professional that attended at their births. Another major strength of our study was the detailed information that we collected regarding the course of their childbirth, including several birth interventions. These data were of great value for evaluating the known group validity of the MADM, MORi and CEQ2.0-. The second strength of the study was that of all eligible women who completed the online survey. Only 2% gave inconsistent answers in the questionnaire and where therefore excluded in the analyses. The third strength of our study was the inclusion of a substantial part (41.2%) of women living in the North of the Netherlands. Compared to other Dutch regions, those women are significantly more likely to be classified in the lowest socio-economic status quartile [41].This is reflected in our included population since 7.6% had obtained a low education level and 17.6% had a monthly income less than €2000. Our study showed that women who differ in socio-economic status are able to complete the three measures. To minimize recall bias, only women who gave birth < 1 year prior were included in the study. It is difficult to determine what time frame between childbirth and conducting the study is best, as there no clear evidence supporting these decisions [42]. Literature does show that studies carried out soon after childbirth has a higher level of positive experiences compared to studies conducted later, as women need time to reflect [43]. However, studies conducted later have a higher chance on recall bias. The current study takes these aspects into account by the < 1 year time range, as it includes both women who recently gave birth and women who had given birth 11-12 months ago.

We are aware that we did not reach women who had a low literacy, and those who did not read Dutch. In total 6.4% of the women had a minority ethnic background, which is an underrepresentation compared to the complete Dutch population. Our study showed that women with a migrant background had lower scores on the MADM, MORi and CEQ, however due to the small sample size of this subgroup, no statistical differences could be captured. Previous studies reported that women who lived in poverty or had a migration background had statistically significantly lower scores on the MADM and MORi [19, 20].

Of our sample, 54.9% started their births in community midwifery care and 82.0% delivered vaginally. Compared with the Dutch population of pregnant women of singletons the corresponding population numbers in 2019 were 50.7, and 74.5%, respectively [44]. Women who give birth vaginally often rate a better birth experience than women who received care had an instrumental birth or caesarean section [45]. Therefore, it is possible there is an overrepresentation of higher scores in this study.

It is likely that the included women were self-selected by choosing to participate in the online voluntary survey. This is a known limitation and can cause selection bias [35]. Women with a negative birth experience may be more prone to take part in a study covering their birth experience than others, which could lead to an overrepresentation of lower scores in the questionnaire.

### Recommendations

Our results regarding the acceptability and clarity of the MADM and MORi indicated a promising uptake and completion of those measures. However, women provided feedback to enhance clarity even more by adding examples to indicate what their involvement could be in e.g. decision making regarding childbirth interventions (e.g. pain relief treatment). Therefore, our first recommendation is to explore with women by using qualitative research if future (small) adaptions are preferred to the original items. A second recommendation would be to evaluate the clarity of the separate items of the CEQ2.0, by either qualitative or quantitative methods. A recommendation for clinical practice would be to use the MADM, MORi and CEQ2.0 as an exit survey or in facility quality improvement programs. These measurement scores could optimize future maternal healthcare since it will help healthcare professionals to be more reflective about their care provision including their communication skills. For maternal health care providers, the results can help to acknowledge women’s autonomy during labour and birth, especially during situations with referrals or complications and when continuity of care is under threat. Women’s right to decision making is an essential aspect of respectful maternity care provision, and should be secured as best as possible in any circumstance in order to achieve a positive birth experience.

## Conclusion

The results of our study showed that the measures MADM, MORi and CEQ2.0 had good psychometric properties in the Dutch setting. Maternity healthcare providers, policy makers and researchers can utilize between these three validated measures for assessing women’s childbirth experiences.

## Supplementary Information

**Additional file 1.**

**Additional file 2.**

## Data Availability

All data and materials will be available from the corresponding author upon any reasonable request.

## Abbreviations

MADM: Mothers Autonomy in Decision Making Scale
MORi: Mothers on Respect Index
CEQ2.0: Childbirth Experience Questionnaire 2.0
