# Supplementary material for: Assessing Dutch women’s experiences of labour and birth: adaptations and psychometric evaluations of the measures Mothers on Autonomy in Decision Making Scale, Mothers on Respect Index, and Childbirth Experience Questionnaire 2.0
Source: BMC Pregnancy Childbirth. 2022 Feb 18;22:134. doi: 10.1186/s12884-022-04445-0 (PMC8857821; doi:10.1186/s12884-022-04445-0)
Supplement: Supplementary file 1 — Additional file 1. [file 12884_2022_4445_MOESM1_ESM.docx]

# Additional file 1

## Dutch versions of MADM, MORi and CEQ2.0

### Vragen over autonomie/regie (Dutch version MADM)

We zijn benieuwd naar je gesprekken met je verloskundige, gynaecoloog of huisarts over de keuzes die je hebt gemaakt rondom de zorg tijdens je bevalling.

LET OP!

Het kan zijn dat je tijdens je bevalling bent doorverwezen en meerdere zorgverleners hebt gezien. Bijvoorbeeld wanneer je door je eigen verloskundige bent overgedragen aan het ziekenhuis tijdens je bevalling en je daar een gynaecoloog en/of klinisch verloskundige hebt gezien. Is dit het geval dan vul je 'ja' in voor zowel je eigen verloskundige en de gynaecoloog en/of klinisch verloskundige. Bij de zorgverlener(s) die je niet hebt gezien vul je 'nee' in. De vragenlijst kan ingevuld worden voor één specifieke zorgverlener die aanwezig was bij de bevalling. Indien er meerdere aanwezig waren, dan kan de MADM meerdere keren worden ingevuld.

Was er een verloskundige (van een verloskundigenpraktijk) aanwezig bij je bevalling?

- Ja
- Nee

Was er een huisarts aanwezig bij je bevalling?

- Ja
- Nee

Was er een klinisch verloskundige (van het ziekenhuis) aanwezig bij je bevalling?

- Ja
- Nee

Was er een gynaecoloog aanwezig bij je bevalling?

- Ja
- Nee

| **Beschrijf je ervaringen over het maken van beslissingen tijdens je bevalling.** | | | | | | |
| --- | --- | --- | --- | --- | --- | --- |
|  | **Volledig oneens** | **Oneens** | **Beetje** **oneens** | **Beetje** **eens** | **Eens** | **Volledig eens** |
| **1. Mijn verloskundige of gynaecoloog vroeg me in hoeverre ik betrokken wilde zijn in het maken van beslissingen.** | 0 | 0 | 0 | 0 | 0 | 0 |
| **2.Mijn verloskundige of gynaecoloog gaf aan dat er verschillende keuze mogelijkheden of opties waren tijdens mijn bevalling.** | 0 | 0 | 0 | 0 | 0 | 0 |
| **3.Mijn verloskundige of gynaecoloog heeft de voordelen en nadelen uitgelegd over verschillende keuze mogelijkheden of opties tijdens mijn bevalling.** | 0 | 0 | 0 | 0 | 0 | 0 |
| **4.Mijn verloskundige of gynaecoloog heeft mij geholpen alle informatie te begrijpen.** | 0 | 0 | 0 | 0 | 0 | 0 |
| **5.Ik kreeg genoeg tijd om de verschillende keuze mogelijkheden grondig tegen elkaar af te wegen.** | 0 | 0 | 0 | 0 | 0 | 0 |
| **6.Ik kon de zorg kiezen die ik zelf het beste vond.** | 0 | 0 | 0 | 0 | 0 | 0 |
| **7.Mijn verloskundige of gynaecoloog respecteerde mijn keuzes.** | 0 | 0 | 0 | 0 | 0 | 0 |

### Scoringsmethode MADM

De antwoord mogelijkheden hebben een 6-punts Likertschaal, lopend van volledig oneens tot volledig mee eens. De antwoord keuzes worden als volgt gecodeerd:

| **Antwoord keuze** | **Gecodeerde waarde** |
| --- | --- |
| Volledig oneens | 1 |
| Oneens | 2 |
| Beetje oneens | 3 |
| Beetje eens | 4 |
| Mee eens | 5 |
| Volledig mee eens | 6 |

De range van de totale MADM score is 7-42. Een hogere score geeft aan dat er meer mogelijkheden ervaren zijn om een actieve rol te spelen en leidende beslissingen te kunnen nemen tijdens de bevalling.

| Sleutel Niveau van autonomie (bij kwartielen) | |
| --- | --- |
| **Totale score** | **Indicatie van respect** |
| 7 - 15 | Hele lage patiënt autonomie |
| 16 - 24 | Lage patiënt autonomie |
| 25 - 33 | Gemiddelde patiënt autonomie |
| 34 - 42 | Hoge patiënt autonomie |

Peters LL et al. Assessing Dutch women’s experiences of labour and birth: Adaptations and psychometric evaluations of the measures Mothers Autonomy in Decision Making Scale, Mothers on Respect Index and Childbirth Experience 2.0, *BMC Pregnancy Childbirth*  (2022), DOI: 10.1186/s12884-022-04445-0.

### Vragen over respect (Dutch version MORi)

We zijn benieuwd naar je gesprekken met je verloskundige, gynaecoloog of huisarts over de keuzes die je maakt rondom zorg tijdens de bevalling.

LET OP!

Het kan zijn dat je tijdens je bevalling bent doorverwezen en meerdere zorgverleners hebt gezien. Bijvoorbeeld wanneer je door je eigen verloskundige bent overgedragen aan het ziekenhuis tijdens je bevalling en je daar een gynaecoloog en/of klinisch verloskundige hebt gezien. Is dit het geval dan vul je 'ja' in voor zowel je eigen verloskundige en de gynaecoloog en/of klinisch verloskundige. Bij de zorgverlener(s) die je niet hebt gezien vul je 'nee' in. De vragenlijst kan ingevuld worden voor één specifieke zorgverlener die aanwezig was bij de bevalling. Indien er meerdere aanwezig waren, dan kan de MORi meerdere keren worden ingevuld.

Was er een verloskundige (van een verloskundigenpraktijk) aanwezig bij je bevalling?

- Ja
- Nee

Was er een huisarts aanwezig bij je bevalling?

- Ja
- Nee

Was er een klinisch verloskundige (van het ziekenhuis) aanwezig bij je bevalling?

- Ja
- Nee

Was er een gynaecoloog aanwezig bij je bevalling?

- Ja
- Nee

| ***Over het geheel genomen tijdens mijn bevalling….*** | | | | | | | | | | | | |
| --- | --- | --- | --- | --- | --- | --- | --- | --- | --- | --- | --- | --- |
|  | | **Volledig oneens** | | **Oneens** | | **Beetje oneens** | | **Beetje eens** | | **Eens** | | **Volledig eens** |
| **1.…voelde ik me op mijn gemak bij het stellen van vragen.** | | 0 | | 0 | | 0 | | 0 | | 0 | | 0 |
| **2…voelde ik me op mijn gemak bij het afwijzen van aangeboden zorg.** | | 0 | | 0 | | 0 | | 0 | | 0 | | 0 |
| **3...voelde ik mij op mijn gemak om de keuzes rondom zorg te accepteren, die werden aanbevolen door mijn verloskundige/**  **gynaecoloog.** | | 0 | | 0 | | 0 | | 0 | | 0 | | 0 |
| **4…voelde ik me onder druk gezet om de keuzes rondom zorg welke werden aanbevolen door mijn verloskundige/**  **gynaecoloog te accepteren.*** | | 0 | | 0 | | 0 | | 0 | | 0 | | 0 |
| **5…ontving ik zorg waarvoor ik zelf heb gekozen.** | | 0 | | 0 | | 0 | | 0 | | 0 | | 0 |
| **6… werden mijn persoonlijke keuzes gerespecteerd.** | | 0 | | 0 | | 0 | | 0 | | 0 | | 0 |
| **7… werd mijn culturele achtergrond gerespecteerd.** | | 0 | | 0 | | 0 | | 0 | | 0 | | 0 |
| ***Ik had het gevoel dat ik tijdens mijn bevalling slecht behandeld werd door mijn verloskundige of gyenacoloog vanwege:*** | | | | | | | | | | | | |
|  | **Volledig oneens** | | **Oneens** | | **Beetje oneens** | | **Beetje eens** | | **Eens** | | **Volledig eens** | |
| **8…Mijn huidskleur, afkomst, culturele achtergrond of taal.*** | 0 | | 0 | | 0 | | 0 | | 0 | | 0 | |
| **9…Mijn seksuele geaardheid en/gender identiteit.*** | 0 | | 0 | | 0 | | 0 | | 0 | | 0 | |
| **10…Mijn aanvullende zorgverzekering of het niet hebben daarvan.*** | 0 | | 0 | | 0 | | 0 | | 0 | | 0 | |
| **11…Een verschil van mening met mijn zorgverleners over de juiste zorg voor mij of mijn baby.*** | 0 | | 0 | | 0 | | 0 | | 0 | | 0 | |
| ***Tijdens mijn bevalling was ik terughoudend met het stellen van vragen of het bespreken van zorgen omdat:*** | | | | | | | | | | | | |
|  | **Volledig oneens** | | **Oneens** | | **Beetje oneens** | | **Beetje eens** | | **Eens** | | **Volledig eens** | |
| **12…De verloskundige/**  **gynaecoloog haast leek te hebben.*** | 0 | | 0 | | 0 | | 0 | | 0 | | 0 | |
| **13…Ik de zorg tijdens de bevalling anders wilde die anders was dan dat de verloskundige of gynaecoloog aanbevolen had.*** | 0 | | 0 | | 0 | | 0 | | 0 | | 0 | |
| **14…Ik het gevoel had dat de verloskundige/**  **gynaecoloog dacht dat ik moeilijk deed.*** | 0 | | 0 | | 0 | | 0 | | 0 | | 0 | |

*Omgedraaid scorende items

### Scoringmethode MORi

De MORi score is een samengestelde somscore. De som van sectie A, B en C wordt eerst per sectie berekend. Daarna wordt de totaalscore berekend door de sectiescore van A, B en C bij op te tellen.

| **Antwoord keuze** | **Gecodeerde waarde** |
| --- | --- |
| Volledig oneens | 1 |
| Oneens | 2 |
| Beetje oneens | 3 |
| Beetje eens | 4 |
| Eens | 5 |
| Volledig eens | 6 |

## De score van negatieve verwoorde stellingen (items 4, 8, 9, 10, 11, 12, 13 en 14) worden omgedraaid (*). De range van de scores is 14-84, waarbij hogere scores meer respectvolle zorg laten zien.

| **Score table** | |
| --- | --- |
| Totaal score sectie A |  |
| Totaal score sectie B |  |
| Totaal score sectie C |  |
| A + B + C = TOTAAL SCORE |  |

| **SLEUTEL Niveau van ervaren respect (in kwartielen)** | |
| --- | --- |
| **Totale score** | **Indicatie van respect** |
| 14-31 | Erg laag respect |
| 32-49 | Laag respect |
| 50-66 | Gemiddeld respect |
| 67-84 | Veel respect |

Peters LL et al. Assessing Dutch women’s experiences of labour and birth: Adaptations and psychometric evaluations of the measures Mothers Autonomy in Decision Making Scale, Mothers on Respect Index and Childbirth Experience 2.0, *BMC Pregnancy Childbirth*  (2022), DOI: 10.1186/s12884-022-04445-0.

### Ervaringen tijdens de bevalling (Dutch version CEQ2.0)

Vragen over ervaringen tijdens de bevalling.

|  | **Helemaal mee eens*** | **Mee eens** | **Mee oneens** | **Helemaal mee oneens** |
| --- | --- | --- | --- | --- |
| 1. **De bevalling ging zoals ik had verwacht.** | 0 | 0 | 0 | 0 |
| 1. **Ik voelde me sterk tijdens de bevalling.** | 0 | 0 | 0 | 0 |
| 1. **Ik voelde me bang tijdens de bevalling.*** | 0 | 0 | 0 | 0 |
| 1. **Ik voelde me flink tijdens de bevalling.** | 0 | 0 | 0 | 0 |
| 1. **Ik was moe tijdens de bevalling.*** | 0 | 0 | 0 | 0 |
| 1. **Ik voelde me blij tijdens de bevalling.** | 0 | 0 | 0 | 0 |
| 1. **Ik had het gevoel dat ik goed omging met de situatie.** | 0 | 0 | 0 | 0 |
| 1. **Ik wilde dat de zorgverleners meer naar me hadden geluisterd tijdens de bevalling.*** | 0 | 0 | 0 | 0 |
| 1. **Ik nam deel in de besluitvorming over mijn zorg en behandeling, zoveel als ik wenste.** | 0 | 0 | 0 | 0 |
| 1. **Mijn partner en ik werden met warmte en respect behandeld.** | 0 | 0 | 0 | 0 |
| 1. **Tijdens de gehele bevalling kreeg ik de informatie die ik nodig had.** | 0 | 0 | 0 | 0 |
| 1. **Ik had meer aanwezigheid van de verloskundige/gynaecoloog tijdens de bevalling gewenst.*** | 0 | 0 | 0 | 0 |
| 1. **Ik had graag meer aanmoediging gehad van de verloskundige/gynaecoloog. *** | 0 | 0 | 0 | 0 |
| 1. **De verloskundige/gynaecoloog droeg bij aan een sfeer van rust tijdens de bevalling.** | 0 | 0 | 0 | 0 |
| 1. **De verloskundige/gynaecoloog hielp me om mijn innerlijke kracht te vinden.** | 0 | 0 | 0 | 0 |
| 1. **Mijn indruk over de medische bekwaamheid van de zorgverleners gaf me een veilig gevoel.** | 0 | 0 | 0 | 0 |
| 1. **Ik heb veel positieve herinneringen aan de bevalling.** | 0 | 0 | 0 | 0 |
| 1. **Ik heb veel negatieve herinneringen aan de bevalling.*** | 0 | 0 | 0 | 0 |
| 1. **Sommige herinneringen aan de bevalling geven me een neerslachtig gevoel.*** | 0 | 0 | 0 | 0 |

*Omgedraaid scorende items

Onderstaande vragen gaan over je ervaringen van je meest recente/laatste bevalling die maximaal een jaar geleden plaatsvond. Het gaat over jouw ervaringen van de ontsluitingsweeën, persweeën en de bevalling.

Kies/omcirkel het cijfer dat het meest relevant is voor de ervaring van de respondent, waarbij 0 = de laagste score aan de linkerkant en 10 = de hoogste score aan de rechterkant:

1. **Over het geheel genomen, hoe pijnlijk vond u de bevalling? ***

| Geen pijn | 0 1 2 3 4 5 6 7 8 9 10 | Ergst denkbare pijn |
| --- | --- | --- |

1. **Over het geheel genomen, in hoeverre had u het gevoel controle te hebben tijdens de bevalling?**

| Totaal geen controle | 0 1 2 3 4 5 6 7 8 9 10 | Complete controle |
| --- | --- | --- |

1. **Over het geheel genomen, hoe veilig voelde u zich tijdens de gehele bevalling?**

| Helemaal niet veilig | 0 1 2 3 4 5 6 7 8 9 10 | Volledig veilig |
| --- | --- | --- |

### Scoringmethode CEQ2.0

De Childbirth Experience Questionnaire versie 2 (CEQ2.0) is aangepast aan de hand van de originele CEQ, ontwikkeld om de beleving van vrouwen van de bevalling en geboorte te studeren. De vragenlijst bevat 22 vragen en stellingen op vier domeinen van geboorte ervaringen: eigen capaciteit, ervaren veiligheid, professionele ondersteuning en participatie.

# Score per item

De antwoord mogelijkheden hebben een 4-punts Likertschaal, lopend van helemaal mee oneens tot helemaal mee eens. De antwoord keuzes worden als volgt gecodeerd:

| **Antwoord keuze** | **Gecodeerde waarde** |
| --- | --- |
| Helemaal mee eens | 4 |
| Mee eens | 3 |
| Mee oneens | 2 |
| Helemaal mee oneens | 1 |

De vragen over bevallingspijn, gevoel van veiligheid en controle (items 20, 21 en 22) worden gescoord op een schaal van 0-10, waarbij 0 de laagste en 10 de hoogste score is. Het getal krijgt daarna de volgende waardes: 0-2=1, 3-5=2, 6-8=3 and 9-10=4.

De score van negatieve verwoorde stellingen (items 3, 5, 8, 12, 13, 18, 19 en 20) worden omgedraaid (*). De range van de scores is van 1-4, waarbij een hogere score meer positieve ervaringen laat zien.

**Berekenen van de sub schaal score**

De items worden per sub schaal (eigen capaciteit, ervaren veiligheid, professionele ondersteuning en participatie) samengevoegd tot schaalscores door de score van de items in elke schaal op te tellen en te delen voor het aantal items in de sub schaal.

**Berekenen van de totale score**

De sub schaalscores (eigen capaciteit, ervaren veiligheid, professionele ondersteuning en participatie) worden daarna bij elkaar opgeteld en gedeeld door vier.

**CEQ2: Domeinen en overzicht items.**

| Item | Item |
| --- | --- |
|  | **Domein: *Eigen capaciteit (8 items)*** |
| 1 | De bevalling ging zoals ik had verwacht |
| 2 | Ik voelde me sterk tijdens de bevalling |
| 4 | Ik voelde me flink tijdens de bevalling |
| 5 | Ik was moe tijdens de bevalling* |
| 6 | Ik voelde me blij tijdens de bevalling |
| 7 | Ik had het gevoel dat ik goed omging met de situatie |
| 20 | Over het geheel genomen, hoe pijnlijk vond u de bevalling?* |
| 21 | Over het geheel genomen, in hoeverre had u het gevoel controle te hebben tijdens de bevalling? |

|  | **Domein: *ervaren veiligheid (6 items)*** |
| --- | --- |
| 3 | Ik voelde me bang tijdens de bevalling* |
| 16 | Mijn indruk over de medische bekwaamheid van de zorgverleners gaf me een veilig gevoel |
| 17 | Ik heb veel positieve herinneringen aan de bevalling |
| 18 | Ik heb veel negatieve herinneringen aan de bevalling* |
| 19 | Sommige herinneringen aan de bevalling geven me een neerslachtig gevoel* |
| 22 | Over het geheel genomen, hoe veilig voelde u zich tijdens de gehele bevalling? |
|  | **Domein: *Proffesionele ondersteuning (5 items)*** |
| 10 | Mijn partner en ik werden met warmte en respect behandeld |
| 12 | Ik had meer aanwezigheid van de verloskundige/gyneacoloog tijdens de bevalling gewenst* |
| 13 | Ik had graag meer aanmoediging gehad van de verloskundige/gyneacoloog* |
| 14 | De verloskundige/gynaecoloog droeg bij aan een sfeer van rust tijdens de bevalling |
| 15 | De verloskundige/gynaecoloog hielp me om mijn innerlijke kracht te vinden |
|  | **Domein: *Participatie (3 items)*** |
| 8 | Ik wilde dat de verloskundige/gynaecoloog meer naar me hadden geluisterd tijdens de bevalling* |
| 9 | Ik nam deel in de besluitvorming over mijn zorg en behandeling, zoveel als ik wenste |
| 11 | Tijdens de gehele bevalling kreeg ik de informatie die ik nodig had |

Peters LL et al. Assessing Dutch women’s experiences of labour and birth: Adaptations and psychometric evaluations of the measures Mothers Autonomy in Decision Making Scale, Mothers on Respect Index and Childbirth Experience 2.0, *BMC Pregnancy Childbirth*  (2022), DOI: 10.1186/s12884-022-04445-0.
