# Supplementary material for: Assessing Dutch women’s experiences of labour and birth: adaptations and psychometric evaluations of the measures Mothers on Autonomy in Decision Making Scale, Mothers on Respect Index, and Childbirth Experience Questionnaire 2.0
Source: BMC Pregnancy Childbirth. 2022 Feb 18;22:134. doi: 10.1186/s12884-022-04445-0 (PMC8857821; doi:10.1186/s12884-022-04445-0)
Supplement: Supplementary file 2 — Additional file 2. [file 12884_2022_4445_MOESM2_ESM.docx]

**Supplementary table: Median (interquartile range) scores on the Mothers Autonomy in Decision Making Scale and Mothers on Respect Index calculated for each specific healthcare professinal who attended at childbirth (n=621)**

|  | | **Community midwife** | **Hospital-based midwife** | **Obstetrician** | **Community midwife and Hospital-based midwife** | **Community midwife and Obstetrician** | **Hospital-based midwife and Obstetrician** | **Community midwife, Hospital-based midwife and Obstetrician** |
| --- | --- | --- | --- | --- | --- | --- | --- | --- |
|  | | ***n*= 238**  **38.3%** | ***n*= 127**  **20.5%** | ***n*= 40**  **6.4%** | ***n*= 47**  **7.6%** | ***n*= 17**  **2.7%** | ***n*= 113**  **18.2%** | ***n*= 39**  **6.3%** |
|  | | **Median (IQR)** | **Median (IQR)** | **Median (IQR)** | **Median (IQR)** | **Median (IQR)** | **Median (IQR)** | **Median (IQR)** |
| **MEASURES** | |  |  |  |  |  |  |  |
| **Mothers Autonomy in Decision Making Scale** | |  |  |  |  |  |  |  |
|  | Community Midwife | 39 (34-42) |  |  | 38 (34-42) ^a^ | 35 (33-39) ^b^ |  | 35 (29-42) ^a^ |
|  | Hospital based midwife |  | 32 (21-36) |  | 29 (17-35) ^a^ |  | 32 (23-35) | 26 (14-38) ^a^ |
|  | Obstetrician |  |  | 31 (23-36) |  | 27 (17-35) ^b^ | 30 (17-35) | 24 (14-35) ^a^ |
| **Mothers on Respect Index** | |  |  |  |  |  |  |  |
|  | Community Midwife | 78 (74-79) |  |  | 75 (72-79) ^a^ | 75 (71-79) ^b^ |  | 77 (71-79) ^a^ |
|  | Hospital based midwife |  | 72 (64-78) |  | 72 (68-75) ^a^ |  | 69 (62-74) ^a^ | 70 (60-76) ^a^ |
|  | Obstetrician |  |  | 73 (632-76) |  | 71 (67-77) ^b^ | 73 (66-77) ^a^ | 75 (63-79) ^a^ |

^a^ Statistical significant difference on the measurement scores between two or three maternal healthcare providers who attended at childbirth (p≤0.05)

^b^ Statistical significant difference on the measurement scores between two maternal healthcare providers could not be calculated due to low numbers
